# The role of health literacy as a factor associated with tooth loss

**DOI:** 10.11606/s1518-8787.2021055003506

**Published:** 2021-12-09

**Authors:** Carla Fabiana Tenani, Manoelito Ferreira Silva, Carolina Matteussi Lino, Maria da Luz Rosário de Sousa, Marília Jesus Batista

**Affiliations:** I Universidade Estadual de Campinas Faculdade de Odontologia de Piracicaba Programa de Pós-Graduação em Odontologia Piracicaba São Paulo Brasil Universidade Estadual de Campinas. Faculdade de Odontologia de Piracicaba. Programa de Pós-Graduação em Odontologia. Piracicaba, São Paulo, Brasil; II Universidade Estadual de Ponta Grossa Faculdade de Odontologia Departamento de Odontologia Ponta Grossa Paraná Brasil Universidade Estadual de Ponta Grossa. Faculdade de Odontologia. Departamento de Odontologia. Ponta Grossa, Paraná, Brasil; III Universidade Estadual de Campinas Faculdade de Odontologia de Piracicaba Departamento de Ciências da Saúde e Odontologia Infantil iracicaba São Paulo Brasil Universidade Estadual de Campinas. Faculdade de Odontologia de Piracicaba. Departamento de Ciências da Saúde e Odontologia Infantil. iracicaba, São Paulo, Brasil; IV Faculdade de Medicina de Jundiaí Departamento de Saúde Coletiva Jundiaí São Paulo Brasil Faculdade de Medicina de Jundiaí. Departamento de Saúde Coletiva. Jundiaí, São Paulo, Brasil

**Keywords:** Oral Health, DMF Index, Health Knowledge, Attitudes, Practice, Health Education, Dental

## Abstract

**OBJECTIVE:**

The objective was to analyze the role of health literacy (HL) as a factor associated with tooth loss among users of the Brazilian Health System with chronic non-communicable diseases.

**METHODS:**

The cross-sectional analytical study was conducted with adult and elderly users chosen at ten Family Health Clinics in a draw in the town of Piracicaba, São Paulo State, Brazil. A questionnaire was applied with sociodemographic data (sex, age, skin color and education), behavioral data (brushing and flossing), determinants in health (type of dental health services and how often) and clinical data (pain). Mouth conditions were collected by intraoral examination of visible dental biofilm and community Pediodontal Index. The systemic clinical conditions (blood glucose, glycated hemoglobin and blood pressure) were extracted from the medical records. The explanatory variable was HL (low, medium and high), measured with the Health Literacy Scale (HLS-14).

**RESULTS:**

The outcome was tooth loss measured by the index of decayed, missing and filled teeth. Logistic regression was performed using a conceptual model for HL (p < 0.05). For the 238 subjects, the mean age was 62.7 years (± 10.55). Tooth loss was associated with HL in regression models adjusted by type of dental service, dental frequency, and dental floss. In the final model, the factors associated with tooth loss are older age (OR = 1,12; 95%CI: 1,07–1,17), a lower education (OR = 3,43; 95%CI: 1,17–10,10), irregular use of dental floss (OR = 4,58; 95%CI: 1.75 in–7,31), irregular use of dental services (n = 2,60; 95% 1,32–5,12), periodontal pocket (> 4 mm) (n = 0,31; 95%CI: 0,01–0,08), having visible dental biofilm (OR = 7,23; 95%CI: 3,19–16,41) and a higher level of blood sugar (glucose) (n = 1,98; 95%CI: 1.00–3,92).

**CONCLUSIONS:**

tooth loss was associated with HL when adjusted by health behaviors; when sociodemographic variables and clinical conditions were included, it was less significant. In the final model, behaviors, determinants in health and clinical conditions were risk indicators of tooth loss, showing the multifactorial nature of this phenomenon.

## INTRODUCTION

Chronic Non-Communicable Diseases (NCDs) are characterized by their multifactorial etiology and are associated with several risk factors^1^. It is estimated that in 2020, 61 million people had diabetes in the Americas alone, and 70.9 million people died worldwide in 2019 as a result of *diabetes mellitus*^3^. Another important risk factor is hypertension, which affected more than a billion people in the American continent alone^4^, in 2019, systolic blood pressure was the main global factor of deaths among individuals over 50 years of age^2^. These numbers lead to the conclusion that strategies to fight and prevent these conditions are urgently needed.

Currently in the Americas, 81% of deaths occur from NCDs^4^; in Brazil, in 2016, it is estimated that 74% of deaths were caused by the same reason^5^. In São Paulo State alone, in 2017, 65% of deaths were due to NCDs^6^, and several other studies show that patients with chronic diseases also demand emergency services and hospitalizations^7,8^.

Another major challenge to the health of the population are mouth conditions^9^, especially tooth loss, which ranked in 2019 as the 22^nd^ largest cause of health deficiency, 31^st^ in prevalence and 56^th^ in incidence^10^. Caries and periodontal disease, in addition to other health behaviors, are the main known risk factors for tooth loss,^11^ but studies show that tooth loss has been associated with systemic changes such as cardiac risk, for example, showing the need for strategies with integral approaches to care.^12^ In this sense, the World Health Organization (WHO) has placed emphasis on health literacy (HL) as an important key to health promotion, as it is considered a measurable and modifiable factor.

Unlike HL, the structural determinants of health are more difficult to modify, literacy can be changed through health promotion interventions, group education, motivational interviews and counseling, thereby increasing autonomy in decision-making. Changes in literacy levels can be measured by using validated instruments of easy application to approach patients, either individually or collectively^13,14^.

Health literacy is the ability to obtain and understand basic information necessary for making health decisions, covering crucial components for seeking well-being and health promotion. For this reason, it is an important marker of inequality^15,16^. Asa research field, HL has been gaining prominence both as an interference factor in health behaviors and conditions^17,18,19^ and in the epidemiological transition with increased NCDs. It is currently very easy to obtain health information, mainly on the internet; however, much of this information is inaccurate, especially because of the indiscriminate spread of fake news, which promote misinformation and affect the health of the population, even threatening lives^20^. In this sense, HL can be of great relevance for determine decision-making in healthcare^15^, even more so when controlled and directed by factors such as age, income, employment, education and skin color. Recent literature shows that individuals with low levels of HL have shorter lifespans, more diseases, cannot use health services and generate more costs to the services^21^.

In light of that, it is relevant to verify the role of HL in tooth loss from a theoretical conceptual model, in the context of NCDs. Thus, the objective of this study was to analyze the role of HL as a factor associated with tooth loss among users of the Brazilian Health System (SUS) with NCDs.

## METHODS

### Study Design and Site

This is a cross-sectional analytical study conducted under the Strengthening the Reporting of Observational Studies in Epidemiology (Strobe) for cross-sectional studies^22^, in the town of Piracicaba, São Paulo State, Brazil, with a random sample among users of Family Health Clinics (FHC) in Primary Health Care (PHC) of the Brazilian Health System (SUS).

### Universe and Sample

According to the 2010 Census, the estimated population of Piracicaba was 364,571 residents in the urban area, and the adult and elderly population was 261,567^23^. The town’s healthcare network had 71 basic health clinics, 51 of which were FHCs^24^.

For the Health Clinics, Morgan’s study (2013) was considered^26^ and out of the 51 FHCs in Piracicaba, the determined number was eight FHCs and four substitute clinics.

For the individuals, the sample size was calculated considering the prevalence of high HL as 50%^25^, 10% error and two delineation effect. The final sample estimated for the study was 238 individuals. Estimating a probable loss, 20% was added, thus totaling 298 individuals.

### Sample Selection

For the selection of Health Clinics, a draw was held of 8 units and 4 more substitutes^26^ in a probabilistic way, considering the number of hypertensive and diabetic patients registered per clinic in the town’s computer system. After two refusals, two substitute FHCs were included, but due to the difficulty in reaching the number of users in some FHCs, another 2 substitutes were included to reach the proposed sample size, totaling at the end 10 FHCs for selection of participants.

When selecting the sample, to compensate for losses, 10 more participants in each of the ten FHCs were added to the sample size through an invitation to participate in the study. Thus, 400 users were selected so that the mininum number (n = 238) could be reached.

The inclusion criteria in the study were: being an adult user (≥ 20 years) registered in the selected FHC; a diagnosis of NCDs (type 2 diabetes and/or systemic arterial hypertension) followed by the clinic; being available to come to the clinic. The exclusion criteria were: exhibit mouth pain or abscess on the day of the interview; refusal to undergo oral clinical examination; having a physical and psychological state (informed by the FHC) that prevented the completion of the examinations and understanding of the questionnaire.

The users were invited to participate in the study by Community Health Agents (personally, during home visits, when coming to the clinic for an appointment or in the HiperDia group). The evaluations took place during the FHC work hours at dates, times and places chosen by the manager himself.

### Data Collection

Data collection was undertaken from July to December 2019 by a dentist who had been previously trained, between May and June 2019, by a “gold standard” Examiner, including theoretical and practical discussions for eight hours, in order to obtain at least 90% agreement for caries, presence of visible dental biofilm and periodontal pocket^27,28^. Intraobserver agreement varied from 90.6% to 100% for dental conditions and periodontal disease, which places it within the reliability standards^29^.

Initially, a pilot collection was performed with users (n = 18) in one of the selected FHCs. When considering that there was no need to change the collection pattern or to adapt the questionnaire, the participants of the pilot study were included in the final sample.

Data collection took place inside the FHC facilities, with questionnaire application, oral clinical examination and collection in clinical records.

A questionnaire was applied with 66 adapted^30^ objective questions in order to obtain data on sociodemographic factors, behavior and determinants in health, HL evaluated using the following data: 14-Item Health Literacy Scale (HLS-14)^31^, translated and validated in Brazil by Batista et al^19^, with 14 questions on a Likert scale (5 points), with the following categories: “strongly disagree”, “disagree”, “neither agree nor disagree”, “agree” and “strongly agree”; and total score from 14 and 70 points, in which higher scores indicate better HL. Questions 1 to 5 are related to the functional dimension and have their score inverted, that is, agreeing with affirmations means having low LS, and the other issues related to communicative literacy (6 to 10) and critical (11 to 14).

The clinical conditions evaluated were the existence of visible dental biofilm (on at least one surface) according to Ainamo and Bay (1975)^27^, index of decayed, lost and filled permanent teeth (CPOD) and community Periodontal Index (CPI)^28^. The participants were examined in the premises of the FHC while seated in chairs, with the aid of natural light, using a *Ball Point* probe and a sterilized clinical mouth mirror, following the criteria set by the WHO^28^. The latest clinical data regarding diabetes and systemic blood pressure were extracted from the medical records of the participants at the FHC.

### Study Variables

**Figure 1:**
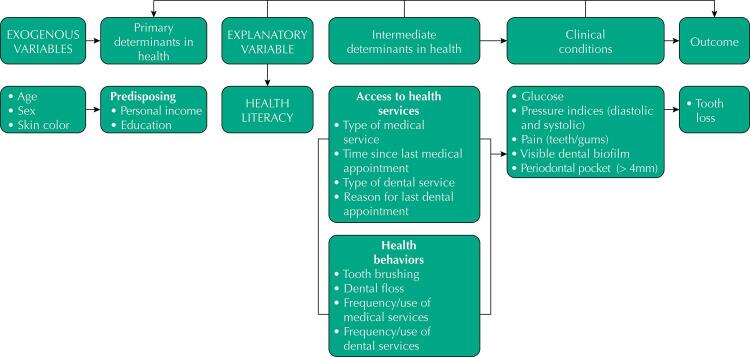
Theoretical conceptual model for health literacy associated with tooth loss.

Health Literacy was considered as an explanatory variable (Figure 1) and was evaluated through the HLS-14 in which the sum of *score* varies from 14 to 70 points, with higher scores indicating higher HL. After the descriptive analysis, HL was divided into thirds: low (0 to 33 points), medium (from 34 to 46 points), and high (> 46 points)^34^.

The variables were selected according to the theoretical conceptual model adapted for the study (Figure 1), categorized as:

Exogenous: age (continuous), sex (female and male) and skin color (white and other “yellow, black or brown”);

Primary determinants in health: personal income (up to one minimum salary, and above one minimum salary) and education (less than 4 years, or up to 4 full years, and 5 years or more – elementary school 1 and 2), with the inclusion of illiterate persons and considering elementary school as a cut-off point for age, in this sample;

Intermediate determinants in health: health-related behaviors, such as tooth brushing (3 or more times a day, and up to 2 times a day), flossing (uses daily, and does not use daily), frequency/use of medical and dental services (once a year or more, and less than once a year);

Access to health services: type of medical or dental service (public or private), time of last medical consultation (less than one year, and more than one year) and reason for dental consultation (routine, pain or need) adapted^30,33^;

Clinical conditions: pain (teeth and/or gums; no pain, and some pain) adapted^30^, visible dental biofilm (yes for at least one surface with biofilm, or not) and periodontal pocket (CPI Code 3 or 4, pouch > 4mm) (yes or not)^28,33^. Glycemic control monitored by fasting blood glucose (altered < 126mg / dl), diabetes *mellitus* (127 mg/dl or more), glycated hemoglobin (HbA1c) (up to 7.0% and 7.1% or more)^34,35^, and SAH whereas normal (systolic [< 130 mmHg] and diastolic [85–89]), and hypertensive patients with (systolic [≥ 140/90mmHg] and diastolic [90–99mmHg or more]), or taking antihypertensive drugs^36^.

The outcome of the study (dependent variable) was tooth loss, with a cutoff point based on the theory of the shortened dental arch, which considers satisfactory the existence of ten pairs of occlusive teeth without aesthetic gaps.^37^ The calculation of missing teeth was performed by codes 4 (tooth missing due to caries) and 5 (tooth missing due to other reasons) of the DMFT index, and the variable tooth loss was categorized into: has 20 or more teeth, has between 19 and 1 tooth, and edental (has no teeth). The third molars were excluded from the examination, so the total tooth loss was having lost 28 teeth.

As shown in Figure 1, a conceptual theoretical model of determinants in health based on Nutbeam was built for the study^38^, considering health literacy, the adapted model of Sørensen^39^ et al. and Martins^40^ et al., for analyzing oral health conditions under the primary determinants and health behaviors^41^.

### Data Analysis

The analysis was carried out on the software Statistical Package for the Social Sciences (SPSS), version 20.0. First, descriptive analyses were performed to obtain the frequency, mean, median, standard deviation and Chi-Square test of the variables collected, based on the theoretical model (Figure 1), with a significance level of 5%.

Then, ordinal logistic regression models were analyzed for the tooth loss condition (3 categories). Logistic regression analysis was performed with a hierarchical approach, according to the model shown in Figure 1. For inclusion in the model, in each block the cut was considered to be p < 0.20 and the significance in the final model was p < 0.05.

The adjustments were: Model 1: regression model with age and education; Model 2: adjusted by type of dental service and HL; Model 3: adjustment for periodontal pocket, tooth/gum pain, visible dental biofilm and blood glucose; Model 4: adjustments of Models 1 and 2 with education, HL and age; Model 5: adjustments of Models 1, 2 and 3, education, HL, age, frequency/dental use and flossing; Model 6: adjustments of Models 1, 2, 4 and 5 with education, HL, age, frequency/dental use, flossing, periodontal pocket, visible dental biofilm and blood glucose.

### Ethical Aspects

The study protocol was submitted and approved by the Research Ethics Committee under CAAE 94104618.7.0000.5418. The Informed Consent Form was previously signed by all participants.

## RESULTS

A total of 238 users with chronic oral diseases followed at ten FHCs participated in this study. Of those, 7.2% (n = 17) of the users had diabetes, 46.6% (n = 111) of them had SAH, and 46.2% (n = 110) had diabetes and SAH. Two users refused to undergo the clinical examination and there was loss of sample due to 162 users not showing up. The response rate was 59.5%, but the desired minimum was achieved.

The mean age of the participants was 62.7 (± 10.55) years, and the majority were women, 69.3% (n = 165). They had lower education 78.5% (n = 187) among the participants and the level of low HL occurred in 33.8% (n = 84), the average level in 36.8% (n = 85) and high level in 29.3% (n = 69).

Low HL was associated with the lower frequency of brushing, irregular use of dental services, irregular flossing, higher prevalence of edentulism, presence of some kind of pain (gum/teeth), systolic blood pressure, periodontal pocket (> 4mm) and visible dental biofilm (Table 1).

**Table 1:** Characteristics of health literacy levels and factors associated with total, p-value (< 0.05), in 238 individuals with chronic diseases, primary health care users in Piracicaba, São Paulo State, Brazil, 2019.

Classification of variables		Classification of Health Literacy (HL^a^)				

		Total	Low HL	Medium HL	High HL	p
			
		n (%)	n (%)	n (%)	n (%)	
Exogenous variables						
Mean age in years (SD)	62.7 (± 10.55)	238 (100)	66.1 (± 8.66)	62.8 (± 9.78)	58.2 (± 12.00)	**< 0.001**
Sex	Female	165 (69.3)	62 (37.6)	56 (33.9)	47 (28.5)	0.518
	Male	73 (30.7)	22 (30.1)	29 (39.8)	22 (30.1)	
Skin color	White	168 (80.0)	59 (35.1)	57 (33.9)	52 (31.0)	0.322
	Other	42 (20.0)	14 (33.3)	19 (45.2)	9 (21.4)	

Primary determinants in health						
Personal income	Above 1 MS^b^	165 (69.3)	57 (34.5)	56 (33.9)	52 (31.5)	0.419
	Up to 1 MS^b^	73 (30.7)	27 (37.0)	29 (39.7)	17 (23.3)	
Education	Less than 4 years	86 (36.1)	38 (44.2)	31 (36.0)	17 (19.8)	**< 0.001**
	Up to 4 full years	101(42.4)	38 (37.6)	40 (39.6)	23 (22.8)	
	5 years or more	51(21.4)	08 (15.7)	14 (27.5)	**29 (56.9)**	

Intermediate determinants of health						

Access to health services						
Type of medical service	Public	212 (89.5)	80 (37.7)	75 (35.4)	57 (26.9)	0.067
	Private	25 (10.5)	04 (16.0)	10 (40.0)	11 (44.0)	
Type of dental service	Public	115(48.7)	32 (27.8)	**52 (45.2)**	31 (27.0)	**0.010**
	Private	121(51.3)	52 (43.0)	33 (27.3)	36 (29.8)	
Reason for dental appointment	Routine	145 (61.2)	52 (35.9)	54 (37.2)	39 (26.9)	0.728
	Pain or need	92 (38.8)	32 (34.8)	31 (33.7)	29 (31.5)	
Time since last medical appointment	Up to 1 year	218 (91.6)	80 (36.7)	75 (34.4)	63 (28.9)	0.259
	More than 1 year	20 (8.4)	04 (20.0)	10 (50.0)	06 (30.0)	

Health behaviors						
Tooth brushing	Up to 2 times/day	103 (43.3)	**47 (45.6)**	29 (28.2)	27 (26.2)	**0.012**
	3 or more times/day	135 (56.7)	37 (27.4)	56 (41.5)	42 (31.1)	
Dental floss	Uses daily	76 (31.9)	11 (14.5)	32 (42.1)	33 (43.4)	**< 0.001**
	Does not use daily	162 (68.1)	**73 (45.1)**	53 (32.7)	36 (22.2)	
Frequency/use of medical services	1 time/year or more (regular use)	181 (76.1)	70 (38.7)	66 (36.5)	45 (24.9)	**0.030**
	- 1 time/year (irregular use)	57 (23.9)	14 (24.6)	19 (33.3)	**24 (42.1)**	
Frequency/use of dental services	+ 1 time/year or more (regular use)	58 (25.2)	08 (13.8)	24 (41.4)	26 (44.8)	**< 0.001**
	1 time/year (irregular use)	172 (74.8)	**75 (46.3)**	59 (34.3)	38 (22.1)	

Clinical conditions						
Tooth loss	Has up to 20 teeth	75 (31.5)	15 (20.0)	24 (32.0)	**36 (48.0)**	**< 0.001**
	Has 1 to 19 teeth	86 (36.1)	33 (38.4)	32 (37.2)	21 (24.4)	
	Edental	77 (32.4)	**36 (46.8)**	29 (37.7)	12 (15.6)	
Pain (teeth/gums)	No pain	156 (66.5)	57 (36.5)	47 (30.1)	52 (33.3)	**0.029**
	Some pain	82 (34.5)	27 (32.9)	**38 (46.3)**	17 (20.7)	
Glucose	Up to 126 dmgl	113 (47.5)	41 (36.3)	37 (32.7)	35 (31.0)	0.693
	127 dmgl or more	125 (52.5)	43 (43.4)	48 (38.4)	34 (27.2)	
Glycated hemoglobin (HbA1c)	Up to 7.0%	92 (38.7)	35 (38.0)	30 (32.6)	27 (29.3)	0.643
	7.1% or more	146 (61.3)	49 (33.6)	55 (37.7)	42 (28.8)	
Systolic blood pressure	Up to 139 mmhg	174 (73.1)	**69 (39.7)**	61 (35.1)	44 (25.3)	**0.036**
	140 mmhg or more	64 (26.9)	15 (23.4)	24 (37.5)	25 (39.1)	
Diastolic blood pressure	Up to 89 mmhg	210 (88.2)	77 (36.7)	70 (33.3)	63 (30.0)	0.110
	90 mmhg or more	28 (11.8)	07 (25.0)	15 (53.6)	06 (21.4)	
Periodontal pocket (> 4mm)	Yes	137 (57.6)	**57 (41.6)**	39 (35.8)	31 (22.6)	**0.017**
	No	101 (42.4)	27 (26.7)	36 (35.6)	38 (37.6)	
Visible dental biofilm	Yes	81 (34.0)	20 (24.7)	30 (37.0)	31 (38.3)	**0.021**
	No	155 (65.1)	**63 (40.6)**	55 (35.5)	37 (23.9)	

Source: prepared by the authors (2020). Chi-square test.

^a^ Health literacy (HL) trichotomized into 3 groups, 1. (above 46 points) ranked as high literacy, 2. (34 to 46 points), medium, and 3. (0 to 38 points) low literacy. HLS - 14 (Suka et al., 2013; Batista et al., 2020).

^b^ Brazilian minimum monthly salary (MS) = R$ 998.00 (Dec / 2019). 3 - For tooth loss, the third molars were excluded considering a total of 28 teeth.

**Figure 2:**
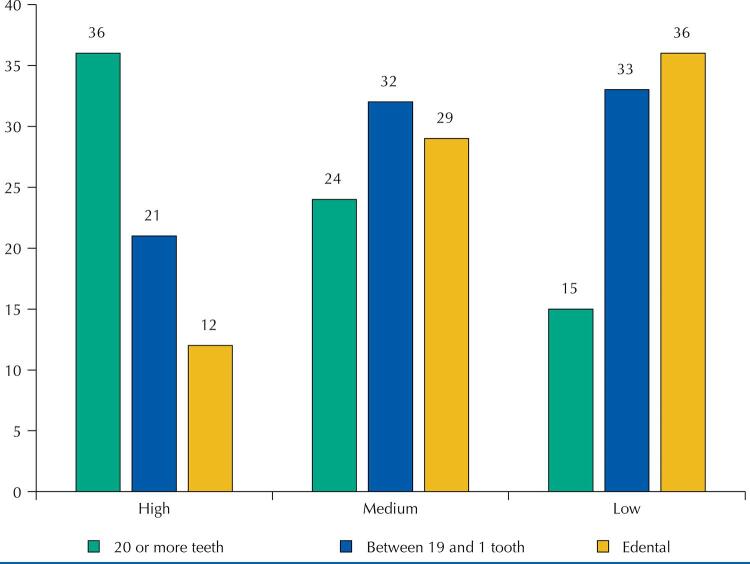
Gradients of tooth loss according to health literacy gradients.

The mean missing teeth in the sample was 14.63 (± 9.36). Figure 2 shows the gradients of tooth loss in relation to the gradients of health literacy: the higher the literacy gradient, the lower the prevalence of edentulism, and the higher prevalence of the presence of 20 or more teeth.

Tooth loss was associated with literacy in Model 2 by mean HL (OR = 2.80; 95%CI: 1.50–5.20) and low HL (OR = 4.70; 95%CI: 2.50–8.82), when adjusted by type of dental service. However, the HL became less significant when other variables were included from Model 3, adjusted for visible periodontal pocket, pain (teeth/gums), visible dental biofilm and blood glucose (Table 3).

**Table 3:** Regression models for the oral condition tooth loss of individuals (n = 238) with chronic diseases, users of the Public Health Service of Piracicaba, São Paulo State, 2019.

VARIABLES		Model 1			Model 2			Model 3			Model 4			Model 5			Model 6		
					
		OR	(95%CI)	p	OR	(95%CI)	p	OR	(95%Cl)	p	OR	(95%CI)	p	OR	(95%CI)	p	OR	(95%CI)	p
**Age**	Mean years (62.7)	1.12	(1.08–1.16)	**0.000**							1.11	(1.08–1.15)	**0.000**	1.10	(1.06–1.14)	**0.000**	1.12	(1.07–1.17)	**0.000**
**Education**	Less than 4 years	6.52	(2.85–14.91)	**0.000**							5.55	(2.37–12.97)	**0.000**	3.18	(1.27–7.93)	**0.013**	3.15	(1.01–9.74)	0.046
	**Up to 4 full years**	6.00	(2.80–12.85)	**0.000**							5.14	(2.35–11.25)	**0.000**	2.90	(1.22–6.93)	**0.016**	3.43	(1.17–10.10)	**0.025**
	5 years or more	1									1			1			1		
**HL**	Lower				4.70	(2.50–8.82)	**0.000**				1.86	(0.92–3.77)	0.083	1.45	(0.68–3.08)	0.326	0.93	(0.37–2.36)	0.894
	Media				2.80	(1.50–5.20)	**0.001**				1.67	(0.83–3.35)	0.146	1.84	(0.88–3.85)	0.104	1.59	(0.65–3.88)	0.308
	High				1.00						1			1			1		
**Use of dental floss**	**Does not use daily**													3.58	(1.75–7.31)	**0.000**	4.88	(1.99–11.95)	**0.001**
	Uses daily													1			1		
**Frequency/use of dental services**	regular use (up to 1 year)				1									1			1		
	**Irregular use (+1 year)**				2.60	(1.32–5.12)	**0.005**							1.94	(0.93–4.03)	0.075	3.15	(1.25–7.95)	**0.015**
**Type of dental service**	Private				1														
	Public				1.38	(0.84–2.25)	0.196												
**Periodontal pocket (>4mm)**	No							1									1		
	**Yes**							0.36	(0.01–0.07)	**0.000**							0.31	(0.01–0.08)	**0.000**
**Oral pain**	No pain							1											
	Some pain							0.54	(0.30–0.96)	0.036									
**Visible dental biofilm**	No							1									1		
	**Yes**							3.84	(2.09–7.04)	**0.000**							7.23	(3.19–16.41)	**0.000**
**Glucose**	Up to 126 dgml							1									1		
	**127 dgml or more**							1.50	(0.87–2.58)	0.138							1.98	(1.00–3.92)	**0.049**

Note: **Model 1:** regression model with age and education; **Model 2:** adjusted by type of dental service and HL; **Model 3:** fit for periodontal pocket, tooth/gum pain, visible dental biofilm and blood glucose; **Model 4:** adjustments of Models 1 and 2 with education, HL and age ; **Model 5:** adjustments of Models 1, 2 and 3, education, HL, age, frequency/use of dental services and flossing; **Model 6:** adjustments of Models 1, 2, 3 and 4 with education, LS, age, frequency/dental use, flossing, periodontal pocket, visible dental biofilm and blood glucose. Multinomial logistic regression.

In the final model, tooth loss was associated with older age(OR = 1,12; 95%CI: 1,07–1,17), lower education level (OR = 3,43; 95%CI: 1,17–10,10), a higher level of blood sugar (glucose) (OR = 1,98; 95%CI: 1,00–3,92), no dental floss use (OR = 4,58; 95%CI: 1,75–7,31), irregular use of dental services (OR = 2,60; 95%CI: 1,32–5,12), periodontal pocket > 4mm (OR = 0,31; 95%CI: 0,01–0,08) and visible dental biofilm (OR = 7,23; 95%CI: 3,19–16,41) (Table 3).

**Table 2:** Characteristics of dental loss and sociodemographic variables access, behavior and health conditions among individuals (n = 238) users with chronic non-communicable diseases, primary health care users in Piracicaba, São Paulo State, Brazil, 2019.

Classification of variables		Classification of Tooth Loss^a^					

		Has 20 teeth or more	Has between 1 and 19 teeth	Edentals	Gross OR	95%CI	p
Exogenous variables							
Mean in years (SD)	62.7 (± 10.55)	54.07 (± 9.03)	64.63 (± 8.24)	68.83 (± 7.84)	1.38	1.10–1.17	**< 0.001**
Sex	Female	55 (33.3)	58 (35.2)	52 (31.5)	1		0.444
	Male	20 (27.4)	28 (38.4)	25 (34.2)	1.22	0.74–2.02	
Skin color	White	52 (31.0)	58 (34.5)	58 (34.5)	1		
	Other	12 (28.6)	16 (38.1)	14 (33.3)	1.03	0.55–1.90	0.938

Primary determinants in health							
Personal Income	Above 1 ^(b)^ MS	63 (38.2)	55 (33.3)	47 (28.5)	1		**0.003**
	Up to 1 ^(b)^ MS	12 (16.4)	31 (42.5)	30 (41.1)	2.18	1.31–3.64	
Education	Less than 4 years	12 (14.0)	30 (34.9)	44 (51.2)	16.22	7.57–34.78	**< 0.001**
	Up to 4 full years	27 (26.7)	43 (42.6)	31 (30.7)	6.88	3.37–14.06	
	5 years or more	36 (70.6)	13 (25.5)	2 (3.9)	1		
Health Literacy	High	36 (52.2)	21 (30.4)	12 (17.4)	4.26	2.91–6.23	**< 0.001**
	Media	24 (28.2)	32 (37.6)	29 (34.1)	2.73	1.88–3.97	**< 0.001**
	Lower	15 (17.9)	33 (39.3)	36 (42.9)	1		

Intermediate determinants of health							

Access to health services							
Type of medical service	Public	62 (29.2)	67 (36.3)	73 (34.4)	2.37	1.09–5.16	0.029
	Private	12 (48.0)	9 (36.0)	4 (16.0)	1		
Type of dental service	Public	30 (26.1)	48 (41.7)	37 (32.2)	1.29	0.81–2.06	0.291
	Private	35 (47.2)	37 (30.6)	39 (32.2)	1		
Reason for dental appointment	Routine	46 (31.7)	47 (32.4)	52 (35.9)	1		
	Pain or need	29 (31.5)	39 (42.4)	24 (26.1)	0.80	0.50–1.30	0.300
Time since last medical appointment	Less than 1 year	71 (32.6)	77 (35.3)	70 (32.7)	1		
	1 time per year or more	4 (20.0)	9 (45.0)	7 (35.0)	3.39	2.04–5.63	**< 0.001**

Health behaviors							
Tooth brushing	Up to 2 times/day	21 (20.4)	34 (33.0)	48 (4.66)	2.91	1.78–4.76	**< 0.001**
	3 or more times/day	54 (40.0)	52 (38.5)	29 (21.5)	1		
Dental floss	Uses daily	46 (60.5)	28 (36.8)	0 (0.0)		1	
	Does not use daily	29 (17.9)	58 (35.8)	75 (43.3)	9.16	5.13–16.37	**< 0.001**
Frequency/use of medical services	1 time/year or more (regular use)	57 (31.5)	63 (34.8)	61 (33.7)	1		
	- 1 time/year (irregular use)	18 (31.6)	23 (40.4)	16 (28.1)	0.88	0.58–1.33	0.537
Frequency/use of dental services	1 time/year or more (regular use)	34 (58.6)	21 (36.2)	3 (5.2)	1		
	- 1 time/year (irregular use)	39 (22.7)	63 (36.6)	70 (40.7)	5.77	1.95–17.10	**0.002**

Clinical conditions	No pain	45 (28.8)	53 (34.0)	58 (37.2)	1		
Pain (teeth/gums)	Some pain	30 (36.6)	33 (40.2)	19 (23.2)	0.61	0.37–1.01	0.054
Glucose	Up to 126 dmgl	38 (33.6)	40 (35.4)	35 (31.0)	1		
	127 dmgl or more	37 (29.6)	46 (36.8)	42 (33.6)	1.17	1.05–1.30	**0.006**
Glycated hemoglobin (HbA1c)	Up to 7.0%	35 (38.0)	27 (29.3)	30 (32.6)	1		
	7.1% or more	40 (27.4)	59 (40.4)	47 (32.2)	1.27	0.55–2.95	0.579
Systolic blood pressure	Up to 139 mmhg	55 (31.6)	64 (36.8)	55 (31.6)	1		
	140 mmhg or more	20 (31.2)	22 (34.4)	22 (34.4)	1.08	0.90–1.29	0.424
Diastolic blood pressure	Up to 89 mmhg	65 (31.0)	80 (38.1)	65 (31.0)	1		
	90 mmhg or more	10 (35.7)	6 (21.4)	12 (42.9)	1.20	0.31–4.63	0.789
Periodontal pocket (> 4mm)	Yes	62 (61.4)	39 (38.6)	0 (0.0)	0.39	0.01–0.47	0.039
	No	13 (9.5)	47 (34.3)	77 (56.2)	1		
Visible dental biofilm	Yes	39 (48.1)	42 (51.9)	0 (0.0)	6.25	0.26–148.10	0.256
	No	34 (21.9)	44 (28.4)	77 (49.7)	1		

^a^ Shortened Arch theory (Armellini and Fraunhofer, 2004).

^b^ Brazilian minimum monthly salary (MS) = R$ 998.00 (Dec / 2019).

## DISCUSSION

Health Literacy was a significant factor associated for tooth loss, even with intermediate determinants in health, access to service and health behaviors, but when adjusted for sociodemographic factors and clinical conditions, it became no statistically significant. It was found that as health literacy gradients increase, edentulism decreases. However, it is known that tooth loss is influenced by multiple factors, therefore, as demonstrated in the final model of this analysis, it was associated with age, education, not flossing, irregular use of dental services, existence of periodontal pocket, visible dental biofilm and glycemic index. It is noteworthy that these same factors, with the exception of glycemic index, were also the factors associated with HL in the bivariate analysis.

Health Literacy has been regarded as an intermediate determinant factor in health behaviors and outcomes^17^and a crucial factor for understanding health information nowadays, leading to health maintenance and recovery^15^, as shown by recent studies^18,42^. The association with intermediate behavioral factors, such as regular flossing, access to services, frequency and type of dental services, has also been observed in the literature^11,43,44^, ratifying the role of HL in health decision-making. Thus, it is evident that HL is an important social determinant to be considered as a strategy for on promoting health and well-being.

The use of model adjustment in this study might indicate that the main outcome in oral health, namely tooth loss, is caused by several risk factors accumulated throughout the individual’s history, therefore health behaviors, tooth brushing, flossing and clinical conditions, such as oral and general diseases, more proximal conditions, end up reducing the impact of HL in this outcome, when these aspects are considered. However, it is observed that, as the health literacy gradient increases, so does edentulism.

Therefore, it is necessary to demonstrate the intermediate role of HL in reducing tooth loss, which is the main result of oral health seen as a global challenge, and in periodontal diseases which, associated with NCDs, can have serious consequences and impact the individual’s quality of life^9^, although the association between tooth loss and HL is still inconclusive in the literature^18,45^.

In this study, tooth loss showed associations with older age due to a cutting effect, that is, related to the historic outcome of Brazil’s public policies in oral health and their impact on the population^14^. Education, another primary health determinant, was also associated with tooth loss, corroborating other findings in which lower education is associated with higher prevalence of tooth loss^46,47^, and established as an indicator of health risk^48^. Sociodemographic, economic and age factors, also found in other studies, are structural factors, therefore difficult to modify^49,50^. Health Literacy is a modifiable factor and is strongly associated with behaviors and aspects associated with tooth loss, although other studies are needed to clarify the association with health outcomes. The WHO highlights that HL is key to the development of health promotion^15^ and an important indicator of social disparities^15^.

The income variable was associated with tooth loss in the univariate analysis; however, when adjusting for education and age, it becomes less significant (it was not associated with HL). This may have occurred due to the collinearity with education and/or the homogeneity of the sample in the socioeconomic aspect, where most earn a minimum salary or more and have up to four years of schooling.

This sample showed associations of tooth loss were found with behaviors such as irregular flossing and use of dental services, variables that impact the increase in the prevalence of tooth loss in individuals with chronic diseases, according to a study conducted in Santa Rita, Paraíba State, Brazil^51^. Regarding clinical conditions, existence of dental biofilm and periodontal pocket, associations were also found in other studies, reinforcing the influence of NCDs on periodontal disease, tooth loss and systemic consequences^52,47^. In this study, individuals who had more teeth present in their mouths had more periodontal pockets, considering the large number of individuals who were edental.

Behaviors are influenced by the determinants and have an impact on chronic diseases, therefore paying attention to the types of determinants of inequities^53^can determine improvements in health. In this case, HL can be a modifying agent empowering individuals and placing them as a protagonist of their health. In short, understanding HL associated with health behaviors becomes a considerable predictor.

The association between high blood glucose and tooth loss pointed out in this study proves that tooth loss is an important indicator of systemic health, as other studies have also pointed out^54,55^. It is even considered as a risk factor for heart disease^12,56^ and rheumatoid arthritis^57^. In addition to potentiating more serious health conditions, this condition can be a predictor of mortality^58^, an extremely relevant fact that reinforces the importance of an integral approach in healthcare^59^.

One limitation of this study was the cross-sectional approach, which did not allow a causal inference. Another aspect to be considered was the homogeneity of the sample, in which all individuals exhibit chronic diseases and the possibility of comorbidities. However, the sample is representative of this portion of the population that needs to be studied and inserted into the health services with a unique understanding and with greater consideration for integral health, since it is the age group in which the most severe oral diseases occur, especially tooth loss^60^, its determinants can cause reflexes in systemic health, therefore requiring rapid attention.

Knowing and identifying associated risk factors can enable better management of health care for this population, and the development of health promotion strategies. Since many health systems in the world do not keep up with the increased burden of NCDs and The Associated health needs of the population^61^, this study is a relevant warning for managers to consider integrality in health, as well as LS, in multiprofessional team work^62^ and in their public policy actions aimed at this population. To this end, this study also shows the use of a tool for measuring the HL that is easy to apply, so that health teams can act to control NCDs

When measurable through instruments used by health professionals (such as the one used in this study), HL will also be important for changing clinical outcomes, and it can be modified through interventions and health promotion actions that increase people’s autonomy in decision-making. Therefore, it is relevant to consider integral healthcare and HL in health promotion policies.

## CONCLUSION

Tooth loss was associated with health literacy when adjusted for health behaviors; when sociodemographic variables and clinical conditions were included, health literacy became no significant. In the final model, behaviors, determinants in health and clinical conditions were risk indicators of tooth loss, which shows the multifactorial nature involved in this phenomenon. Therefore, future studies aimed at understanding tooth loss and approaching health literacy and integral healthcare are encouraged.
