# Supplementary material for: The role of health literacy as a factor associated with tooth loss
Source: Rev Saude Publica. 2021 Dec 9;55:116. doi: 10.11606/s1518-8787.2021055003506 (PMC8664067; doi:10.11606/s1518-8787.2021055003506)
Supplement: Translation

# Translation: O papel da literacia em saúde como fator associado às perdas dentárias

**Authors:** Carla Fabiana Tenani, Manoelito Ferreira Silva, Carolina Matteussi Lino, Maria da Luz Rosário de Sousa, Marília Jesus Batista

**Affiliations:** I Brasil Universidade Estadual de Campinas. Faculdade de Odontologia de Piracicaba. Programa de Pós-Graduação em Odontologia. Piracicaba, São Paulo, Brasil; II Brasil Universidade Estadual de Ponta Grossa. Faculdade de Odontologia. Departamento de Odontologia. Ponta Grossa, Paraná, Brasil; III Brasil Universidade Estadual de Campinas. Faculdade de Odontologia de Piracicaba. Departamento de Ciências da Saúde e Odontologia Infantil. Piracicaba, São Paulo, Brasil; IV Brasil Faculdade de Medicina de Jundiaí. Departamento de Saúde Coletiva. Jundiaí, São Paulo, Brasil

**Keywords:** Saúde Bucal, Índice CPO, Conhecimentos, Atitudes e Prática em Saúde, Educação em Saúde Bucal

## Abstract

**OBJETIVO:**

O objetivo foi analisar o papel da Literacia em Saúde (LS) como fator associado às perdas dentárias entre usuários do Sistema Único de Saúde com doenças crônicas não transmissíveis.

**MÉTODOS:**

O estudo transversal e analítico foi conduzido com usuários adultos e idosos selecionados em dez Unidades de Saúde da Família sorteadas, em Piracicaba – SP, Brasil. Foi aplicado um questionário com dados sociodemográficos (sexo, idade, cor da pele e escolaridade), comportamentais (escovação e uso de fio dental), determinantes em saúde (tipo e frequência de uso de serviço de saúde médico e odontológico) e clínica (dor). As condições bucais foram coletadas por exame intrabucal do biofilme dental visível e Índice Pediodontal Comunitário. As condições clínicas sistêmicas (glicemia, hemoglobina glicada e pressóricas) foram extraídas dos prontuários. A variável explanatória foi a LS (baixa, média e alta), medida pelo *Health Literacy Scale* (*HLS-14).*

**RESULTADOS:**

O desfecho foi à perda dentária medida pelo Índice de dentes permanentes cariados, perdidos e obturados. Foi realizada regressão logística com uso de um modelo conceitual para a LS (p < 0,05). Para os 238 indivíduos, a média de idade foi 62,7 anos (± 10,55). A perda dentária esteve associada à LS nos modelos de regressão ajustados por tipo de serviço odontológico, frequência odontológica e uso de fio dental. No modelo final, a perda dentária teve como fatores associados a maior idade (OR = 1,12; IC95% 1,07–1,17), menor escolaridade (OR = 3,43; IC95% 1,17–10,10), ao uso irregular de fio dental (OR = 4,58; IC95% 1,75–7,31), uso irregular do serviço odontológico (OR = 2,60; IC95% 1,32–5,12), bolsa periodontal (> 4mm) (OR = 0,31; IC95% 0,01–0,08), ter biofilme dental visível (OR = 7,23; IC95% 3,19–16,41) e maior índice de glicemia (OR = 1,98; IC95% 1,00–3,92).

**CONCLUSÕES:**

A perda dentária esteve associada à LS quando ajustada por comportamentos em saúde, a partir da inclusão das variáveis sociodemográficas e condições clínicas ela perdeu a significância. No modelo final, comportamentos, determinantes em saúde e condições clínicas foram indicadores de risco da perda dentária, demonstrando a multifatorialidade envolvida neste fenômeno.

## INTRODUÇÃO

As Doenças Crônicas Não Transmissíveis (DCNT) caracterizam-se por sua etiologia multifatorial e estão associadas a diversos fatores de risco^1^. Estima-se que, em 2020, só nas Américas, 61 milhões de pessoas tinham diabetes e, em todo o mundo, 70,9 milhões de pessoas morreram no ano de 2019, em decorrência da *diabetes mellitus*^3^. Outro fator de risco importante é a hipertensão, que afetou mais de um bilhão de pessoas só no continente americano^4^, em 2019, a pressão arterial sistólica foi o principal fator global de óbitos entre indivíduos acima de 50 anos^2^. Esses números levam à constatação da urgente necessidade de estratégias para o enfrentamento e prevenção dessas condições.

Atualmente, nas Américas, 81% das mortes ocorrem por DCNT^4^, no Brasil, em 2016, estima-se que 74% das mortes foram causadas pelo mesmo motivo^5^. Só em São Paulo, em 2017, 65% das mortes em todo o estado foram em decorrência de DCNT^6^, e diversos outros estudos demonstram a procura de pacientes com doenças crônicas por serviços emergenciais e hospitalizações^7,8^.

Outro grande desafio à saúde da população são as condições bucais^9^, principalmente a perda dentária, classificada em 2019 como a 22ª maior causa de deficiência em saúde, 31ª em prevalência e 56ª em incidência^10^. A cárie e a doença periodontal, além de outros comportamentos em saúde, são os principais fatores de risco conhecidos para a perda dentária^11^, no entanto, estudos demonstram que perdas dentárias têm sido associadas a alterações sistêmicas, como risco cardíaco, por exemplo, evidenciando a necessidade de estratégias com abordagens integrais do cuidado^12^, nesse sentido, a Organização Mundial da Saúde (OMS) tem destacado a Literacia em Saúde (LS) como uma chave importante para promoção da saúde, considerada um fator mensurável e modificável.

Ao contrário da LS, os determinantes estruturais de saúde são mais difíceis de serem modificados, a literacia pode ser alterada por meio de intervenções de promoção de saúde, educação em grupo, entrevistas motivacionais e aconselhamentos, aumentando a autonomia nas tomadas de decisão. A modificação dos níveis de literacia pode ser mensurada por meio de instrumentos validados de fácil aplicação para abordar o paciente, individual ou coletivamente^13,14^.

A LS é a capacidade de obter e compreender informações básicas necessárias para tomar decisões em saúde, abrangendo componentes fundamentais na busca por bem-estar e na promoção da saúde, mostrando-se um importante marcador de desigualdade^15,16^. Como campo de pesquisa a LS vem ganhando destaque, seja como fator de interferência em comportamentos e condições de saúde^17,18,19^, seja na transição epidemiológica com aumento das DCNT. Há, atualmente, muita facilidade em se obter informações de saúde, principalmente por meio da internet, porém, muitas dessas informações são errôneas, principalmente graças à disseminação indiscriminada de notícias falsas, que promovem desinformação e afetam a saúde da população, inclusive ameaçando vidas^20^. Nesse sentido, a LS pode ser de grande relevância para determinar a tomada de decisões em saúde^15^, ainda mais quando controlada e direcionada por fatores como idade, renda, emprego, nível educacional e cor da pele.. A literatura recente revela que indivíduos com níveis baixos de LS têm menos tempo de vida, mais carga de doenças, incapacidade de uso de serviço de saúde e geram mais custos aos serviços^21^.

Diante do exposto, é relevante verificar o papel da LS nas perdas dentárias, a partir de um modelo conceitual teórico, no contexto das DCNT. Assim, o objetivo deste estudo foi analisar o papel da LS como fator associado às perdas dentárias entre usuários do Sistema Único de Saúde (SUS) portadores de DCNT.

## MÉTODOS

### Desenho e Local do Estudo

Estudo transversal e analítico, realizado sob o *Strengthening the Reporting of Observational Studies in Epidemiology* (STROBE) para estudos transversais^22^, na cidade de Piracicaba, São Paulo, Brasil, com amostra aleatória entre usuários de Unidades de Saúde da Família (USF) na Atenção Primária em Saúde (APS) do Sistema Único de Saúde (SUS).

### Universo e Amostra

Segundo o Censo em 2010, a população estimada de Piracicaba era de 364.571 residentes na área urbana e a população adulta e idosa correspondia a 261.567^23^. A rede municipal de saúde contava com 71 Unidades Básicas de Saúde, sendo 51 USF^24^.

Para as Unidades de Saúde, considerou-se o estudo de Morgan (2013)^26^ e foi determinado o número de 8 USF e quatro suplentes, dentre as 51 USF de Piracicaba.

Para os indivíduos, o tamanho da amostra foi calculado considerando a prevalência da alta LS como 50%^25^, erro de 10% e efeito de delineamento dois. A amostra final estimada para o estudo foi de 238 indivíduos. Estimando uma provável perda, foi acrescentado 20% chegando ao total de 298 indivíduos.

### Seleção da amostra

Para a seleção das Unidades de Saúde, realizou-se um sorteio de 8 Unidades e mais 4 suplentes^26^, de forma probabilística, considerando o número de hipertensos e diabéticos cadastrados por Unidade no sistema informatizado do município. Após duas recusas, incluíram-se duas USF suplentes sorteadas, porém, devido à dificuldade em atingir o número de usuários em algumas USF, foram incluídas outras 2 suplentes para alcançar o tamanho amostral proposto, totalizando ao final 10 USF para a seleção dos participantes.

Na seleção da amostra, para compensar perdas foi adicionado ao tamanho amostral 10 participantes a mais em cada das dez USF, por meio de convite à participação no estudo. Assim, foram selecionados 400 usuários, para atingir o mínimo de (n = 238).

Os critérios de inclusão no estudo foram: ser usuário adulto (≥ 20 anos) cadastrado nas USF sorteadas; diagnóstico de DCNT (diabetes tipo 2 e/ou hipertensão arterial sistêmica) acompanhado pela Unidade; ter disponibilidade para comparecer a Unidade. Já os critérios para a exclusão foram: apresentar dor bucal ou abcesso no dia da entrevista; recusar o exame clínico bucal; possuir estado físico e psicológico (informados pela USF) que impedissem a realização dos exames e compreensão do questionário.

A participação dos usuários se deu por meio de convite, realizado por Agentes Comunitárias de Saúde (pessoalmente, via visitas domiciliares, comparecimento em consulta ou grupo HiperDia). As avaliações ocorreram durante o funcionamento das USF, em datas, horários e locais estabelecidos pelo próprio gestor.

### Coleta de Dados

A coleta de dados foi realizada de julho a dezembro de 2019, por uma cirurgiã-dentista, treinada previamente, entre maio e junho de 2019, por uma examinadora “padrão-ouro”, incluindo discussões teóricas e práticas por oito horas, a fim de obter pelo menos 90% de concordância para cárie, presença de biofilme dental visível e bolsa periodontal^27,28^. A concordância intraobservador variou de 90,6% a 100% para as condições dentárias e doença periodontal, estando dentro dos padrões de confiabilidade^29^.

Inicialmente foi realizada uma coleta piloto com usuários (n = 18) em uma das USF sorteadas. Ao considerar que não houve necessidade de alterações no padrão de coleta e nem adaptação do questionário, os participantes do estudo piloto fizeram parte da amostra final.

A coleta de dados aconteceu nas dependências das USF, com aplicação de questionário, realização de exame clínico bucal e coleta em prontuários clínicos.

Foi aplicado um questionário, com 66 perguntas objetivas adaptadas^30^, para se obter os dados sobre os fatores sociodemográficos, comportamento e determinantes em saúde A LS foi avaliada utilizando o *14-Item Health Literacy Scale (HLS-14)*^31^, traduzido e validado no Brasil por Batista et al^19^, com 14 questões em escala tipo Likert (5 pontos), com as categorias: “discordo muito”, “discordo”, “nem concordo nem discordo”, “concordo” e “concordo muito”; e pontuação total variando entre 14 e 70 pontos, em que maiores pontuações indicam melhor LS. As questões de 1 a 5 são relacionadas à dimensão funcional e têm seu score invertido, ou seja, concordar com as afirmativas significa possuir baixa LS, e as demais questões relacionadas à literacia comunicativa (6 a 10) e crítica (11 a 14).

As condições clínicas avaliadas foram a presença de biofilme dental visível (em pelo menos uma superfície) de acordo com Ainamo e Bay (1975)^27^, índice de dentes permanentes Cariados, Perdidos e Obturados (CPOD) e Índice Periodontal Comunitário (CPI)^28^. Os participantes foram examinados nas dependências das USF, sentados em cadeiras, com auxílio de luz natural, utilizando sonda tipo “*Ball Point*” e espelho bucal clínico, estéreis, segundo os critérios estabelecidos pela OMS^28^. Os dados clínicos referentes à diabetes e pressão arterial sistêmica mais recentes foram extraídos dos prontuários dos participantes nas USF.

### Variáveis de Estudo

A LS foi considerada como variável explanatória (Figura 1), avaliada através do *HLS-14* em que a soma do *score* varia entre 14 e 70 pontos, com pontuações mais altas indicando maior LS. A LS foi dividida, após análise descritiva, nos tercis em: baixa (0 a 33 pontos), média (de 34 a 46 pontos), e alta (> 46 pontos)^34^.

**Figura 1:** Modelo teórico conceitual para literacia em saúde associada às perdas dentárias.

As variáveis foram selecionadas de acordo com o modelo conceitual teórico adaptado para o estudo (Figura 1), categorizadas em:

Exógenas: idade (contínua), sexo (mulher e homem) e cor da pele (branca e outras “amarela, preta ou parda”);

Determinantes primários em saúde: renda pessoal (até um salário mínimo, e acima de um salário mínimo) e escolaridade (menos que 4 anos, ou até os 4 anos completos, e 5 anos ou mais – ensino fundamental 1 e 2), com inclusão de analfabetos e considerando o ensino fundamental como ponto de corte para a idade, desta amostra;

Determinantes intermediários em saúde: comportamentos relacionados à saúde, como escovação dental (3 ou mais vezes/dia, e até 2 vezes/dia), fio dental (usa diariamente, e não usa diariamente), frequência/uso de serviço médico e odontológico (uma vez ao ano ou mais, e menos que uma vez ao ano);

Acesso a serviços de saúde: tipo de serviço médico ou odontológico (público ou privado), tempo da última consulta médica (menos de um ano, e mais de um ano) e motivo da consulta odontológica (rotina, dor ou necessidade) adaptados^30,33^;

Condições clínicas: dor (dentes e/ou gengivas; sem dor, e alguma dor) adaptado^30^, biofilme dental visível (sim para pelo menos uma superfície com biofilme, ou não) e bolsa periodontal (CPI código 3 ou 4, bolsa > 4mm) (sim ou não)^28,33^. O controle glicêmico monitorado por glicemia de jejum (alterada < 126mg/dl), diabetes *mellitus* (127 mg/dl ou mais), hemoglobina glicada (HbA1c) (até 7,0%, e 7,1% ou mais)^34,35^, e HAS considerando normal (sistólica [< 130 mmHg] e diastólica [85–89]), e hipertensos com (sistólica [≥ 140/90mmHg] e diastólica [90–99mmHg ou mais]), ou em uso de medicação anti-hipertensiva^36^.

O desfecho do estudo (variável dependente) foi à perda dentária, cujo ponto de corte foi baseado na teoria do arco dentário reduzido, que considera satisfatória a presença de dez pares de dentes ocludentes sem lacunas estéticas.^37^ O cálculo de dentes perdidos foi realizado pelo código 4 (dente perdido por cárie) e 5 (dente perdido por outros motivos) do CPOD, e a variável perda dentária foi categorizada em: tem 20 ou mais dentes presentes, tem entre 19 e 1 dentes, e edêntulos (nenhum dente presente). Os terceiros molares foram excluídos do exame, por isso a perda dentária total foi ter perdido 28 dentes.

Conforme ilustrado na Figura 1, foi construído para o estudo um modelo teórico conceitual de determinantes da saúde baseado em Nutbeam^38^, considerando a literacia em saúde, o modelo adaptado de Sørensen^39^ et al. e Martins^40^ et al., para análise das condições de saúde bucal sob os determinantes primários e os comportamentos de saúde^41^.

### Análise dos dados

A análise foi realizada no programa *Statistical Package for the Social Sciences* (SPSS), versão 20.0. Primeiramente, realizaram-se as análises descritivas para obter a frequência, média, mediana, desvio-padrão e teste Qui-Quadrado das variáveis coletadas com base no modelo teórico (Figura 1), com nível de significância de 5%.

Posteriormente foi feita a análise de modelos de regressão logística ordinal para a condição perdas dentárias (3 categorias). Realizou-se análises de regressão logística com abordagem hierárquica, segundo o modelo apresentado na Figura 1. Para a inclusão no modelo, em cada bloco foi considerado o corte de p < 0,20 e a significância no modelo final foi p < 0,05.

Os ajustes foram: Modelo 1: modelo de regressão com idade e escolaridade; Modelo 2: ajustado por tipo de serviço odontológico e LS; Modelo 3: ajuste para bolsa periodontal, dor dentes/gengivas, biofilme dental visível e glicemia; Modelo 4: ajustes dos Modelos 1 e 2 com escolaridade, LS e idade; Modelo 5: ajustes dos Modelos 1, 2 e 3,escolaridade, LS, idade, frequência/uso odontológico e uso de fio dental; Modelo 6: ajustes dos Modelos 1, 2, 4 e 5 com escolaridade, LS, idade, frequência/uso odontológico, uso de fio dental, bolsa periodontal, biofilme dental visível e glicemia.

### Aspectos Éticos

O protocolo do estudo foi submetido e aprovado pelo Comitê de Ética em Pesquisa sob o CAAE 94104618.7.0000.5418. O Termo de Consentimento Livre e Esclarecido foi assinado previamente por todos os participantes.

## RESULTADOS

Participaram desta pesquisa 238 usuários portadores das doenças crônicas bucais acompanhados em dez USF, sendo 7,2% (n = 17) dos usuários tinham diabetes, 46,6% (n = 111) deles tinham HAS, e 46,2% (n = 110) apresentavam diabetes e HAS. Dois usuários se recusaram a realizar o exame clínico e houve uma perda amostral, devido ao não comparecimento, de 162 usuários. A taxa de resposta foi 59,5%, porém, atingiu-se o mínimo pretendido.

A média de idade dos participantes foi de 62,7(± 10,55) anos, com maioria de mulheres, 69,3% (n = 165). Apresentaram menor escolaridade 78,5% (n = 187) dos participantes e o nível de baixa LS ocorreu em 33,8% (n = 84), o de média em 36,8% (n = 85) e alta em 29,3% (n = 69).

A baixa LS foi associada a menor frequência de escovação, uso irregular de serviço odontológico, uso irregular de fio dental, maior prevalência de edentulismo, presença de alguma dor (gengiva/dentes), pressão arterial sistólica, bolsa periodontal (> 4mm) e biofilme dental visível (Tabela 1).

**Tabela 1:** Características dos níveis de literacia em saúde e fatores associados com total, valor de p (< 0,05), em 238 indivíduos com doenças crônicas, usuários da Atenção Primária em Saúde em Piracicaba, SP, Brasil, 2019.

Classificação das variáveis		Classificação da Literacia em Saúde (LS^a^)				

		Total	Baixa LS	Média LS	Alta LS	p
			
		n (%)	n (%)	n (%)	n (%)	
Variáveis exógenas						
Idade média em anos (DP)	62,7 (± 10,55)	238 (100)	66,1 (±8,66)	62,8 (±9,78)	58,2 (±12,00)	**< 0,001**
Sexo	Mulher	165 (69,3)	62 (37,6)	56 (33,9)	47 (28,5)	0,518
	Homem	73 (30,7)	22 (30,1)	29 (39,8)	22 (30,1)	
Cor da pele	Branca	168 (80,0)	59 (35,1)	57 (33,9)	52 (31,0)	0,322
	Outra	42 (20,0)	14 (33,3)	19 (45,2)	9 (21,4)	

Determinantes primários em saúde						
Renda Pessoal	Acima de 1 ^(b)^ SM	165 (69,3)	57 (34,5)	56 (33,9)	52 (31,5)	0,419
	Até 1 ^(b)^ SM	73 (30,7)	27 (37,0)	29 (39,7)	17 (23,3)	
Escolaridade	Menos que 4 anos	86 (36,1)	38 (44,2)	31 (36,0)	17 (19,8)	**< 0,001**
	Até 4 anos completos	101(42,4)	38 (37,6)	40 (39,6)	23 (22,8)	
	5 anos ou mais	51(21,4)	08 (15,7)	14 (27,5)	**29 (56,9)**	

Determinantes intermediários em saúde						

Acesso a serviços de saúde						
Tipo de serviço médico	Público	212 (89,5)	80 (37,7)	75 (35,4)	57 (26,9)	0,067
	Privado	25 (10,5)	04 (16,0)	10 (40,0)	11 (44,0)	
Tipo de serviço odontológico	Público	115(48,7)	32 (27,8)	**52 (45,2)**	31 (27,0)	**0,010**
	Privado	121(51,3)	52 (43,0)	33 (27,3)	36 (29,8)	
Motivo da consulta odontológica	Rotina	145 (61,2)	52 (35,9)	54 (37,2)	39 (26,9)	0,728
	Dor ou necessidade	92 (38,8)	32 (34,8)	31 (33,7)	29 (31,5)	
Tempo da última consulta médica	Até 1 ano	218 (91,6)	80 (36,7)	75 (34,4)	63 (28,9)	0,259
	Mais de 1 ano	20 (8,4)	04 (20,0)	10 (50,0)	06 (30,0)	

Comportamentos em saúde						
Escovação	Até 2 vezes/dia	103 (43,3)	**47 (45,6)**	29 (28,2)	27 (26,2)	**0,012**
	3 ou mais vezes/dia	135 (56,7)	37 (27,4)	56 (41,5)	42 (31,1)	
Fio dental	Usa diariamente	76 (31,9)	11 (14,5)	32 (42,1)	33 (43,4)	**< 0,001**
	Não usa diariamente	162 (68,1)	**73 (45,1)**	53 (32,7)	36 (22,2)	
Frequência/uso de serviço médico	1 vez/ano ou mais (uso regular)	181 (76,1)	70 (38,7)	66 (36,5)	45 (24,9)	**0,030**
	- 1 vez/ano (uso irregular)	57 (23,9)	14 (24,6)	19 (33,3)	**24 (42,1)**	
Frequência/uso de serviço odontológico	+ 1 vez/ano ou mais (uso regular)	58 (25,2)	08 (13,8)	24 (41,4)	26 (44,8)	**< 0,001**
	1 vez/ano (uso irregular)	172 (74,8)	**75 (46,3)**	59 (34,3)	38 (22,1)	

Condições clínicas						
Perda dentária	Tem até 20 dentes	75 (31,5)	15 (20,0)	24 (32,0)	**36 (48,0)**	**< 0,001**
	Tem 1 a 19 dentes	86 (36,1)	33 (38,4)	32 (37,2)	21 (24,4)	
	Edêntulo	77 (32,4)	**36 (46,8)**	29 (37,7)	12 (15,6)	
Dor (dentes/gengivas)	Sem dor	156 (66,5)	57 (36,5)	47 (30,1)	52 (33,3)	**0,029**
	Alguma dor	82 (34,5)	27 (32,9)	**38 (46,3)**	17 (20,7)	
Glicemia	Até 126 dmgl	113 (47,5)	41 (36,3)	37 (32,7)	35 (31,0)	0,693
	127 dmgl ou mais	125 (52,5)	43 (43,4)	48 (38,4)	34 (27,2)	
Hemoglobina Glicada (HbA1c)	Até 7,0%	92 (38,7)	35 (38,0)	30 (32,6)	27 (29,3)	0,643
	7,1% ou mais	146 (61,3)	49 (33,6)	55 (37,7)	42 (28,8)	
Pressão Arterial Sistólica	Até 139 mmhg	174 (73,1)	**69 (39,7)**	61 (35,1)	44 (25,3)	**0,036**
	140 mmhg ou mais	64 (26,9)	15 (23,4)	24 (37,5)	25 (39,1)	
Pressão Arterial Diastólica	Até 89 mmhg	210 (88,2)	77 (36,7)	70 (33,3)	63 (30,0)	0,110
	90 mmhg ou mais	28 (11,8)	07 (25,0)	15 (53,6)	06 (21,4)	
Bolsa periodontal (> 4mm)	Sim	137 (57,6)	**57 (41,6)**	39 (35,8)	31 (22,6)	**0,017**
	Não	101 (42,4)	27 (26,7)	36 (35,6)	38 (37,6)	
Biofilme dental visível	Sim	81 (34,0)	20 (24,7)	30 (37,0)	31 (38,3)	**0,021**
	Não	155 (65,1)	**63 (40,6)**	55 (35,5)	37 (23,9)	

Fonte: elaborada pelos autores (2020). Teste do Qui-quadrado.

^a^ Literacia em Saúde (LS) tricotomizada em 3 grupos, 1. (acima de 46 pontos) classificado em alta literacia, 2. (de 34 a 46 pontos), média, e 3. (0 a 38 pontos) baixa literacia. HLS-14 (Suka et al., 2013; Batista et al., 2020).

^b^ Salário mínimo (SM) brasileiro = R$ 998.00 (Dez / 2019). 3. Para perda dentária foram excluídos os terceiros molares considerando o total de 28 dentes.

A média de dentes perdidos na amostra foi de 14,63 (± 9,36). Na Figura 2 pode-se observar os gradientes de perdas dentárias em relação aos gradientes de literacia em saúde, quanto maior o gradiente de literacia, menor a prevalência de edentulismo, e maior prevalência da presença de 20 ou mais dentes.

**Figura 2:** Gradientes da perda dentária de acordo com os gradientes de literacia em saúde.

A perda dentária esteve associada à literacia no Modelo 2 pela média LS (OR = 2,80; IC95% 1,50–5,20) e baixa LS (OR = 4,70; IC95% 2,50–8,82), quando ajustadas por tipo de serviço odontológico. Porém, a LS perdeu a significância quando foram incluídas outras variáveis a partir do Modelo 3, ajustado por bolsa periodontal visível, dor (dentes/gengivas), biofilme dental visível e glicemia (Tabela 2).

**Tabela 2:** Características das perdas dentárias e variáveis sociodemográficas acesso, comportamento e condições de saúde entre indivíduos (n = 238) usuários portadores de doenças crônicas não transmissíveis, usuários da Atenção Primária em Saúde em Piracicaba, SP, Brasil, 2019.

Classificação das variáveis		Classificação das Perdas Dentáriasa					

		Tem 20 dentes ou mais	Tem entre 1 e 19 dentes	Edêntulos	OR Bruto	IC95%	p
Variáveis exógenas							
Média em anos (DP)	62,7 (±10,55)	54,07 (±9,03)	64,63 (±8,24)	68,83 (±7,84)	1,38	1,10–1,17	**< 0,001**
Sexo	Mulher	55 (33,3)	58 (35,2)	52 (31,5)	1		0,444
	Homem	20 (27,4)	28 (38,4)	25 (34,2)	1,22	0,74–2,02	
Cor da pele	Branca	52 (31,0)	58 (34,5)	58 (34,5)	1		
	Outra	12 (28,6)	16 (38,1)	14 (33,3)	1,03	0,55–1,90	0,938
	
Determinantes primários em saúde							
Renda Pessoal	Acima de 1 ^(b)^ SM	63 (38,2)	55 (33,3)	47 (28,5)	1		**0,003**
	Até 1 ^(b)^ SM	12 (16,4)	31 (42,5)	30 (41,1)	2,18	1,31–3,64	
Escolaridade	Menos que 4 anos	12 (14,0)	30 (34,9)	44 (51,2)	16,22	7,57–34,78	**< 0,001**
	Até 4 anos completos	27 (26,7)	43 (42,6)	31 (30,7)	6,88	3,37–14,06	
	5 anos ou mais	36 (70,6)	13 (25,5)	2 (3,9)	1		
Literacia em Saúde	Alta	36 (52,2)	21 (30,4)	12 (17,4)	4,26	2,91–6,23	**< 0,001**
	Média	24 (28,2)	32 (37,6)	29 (34,1)	2,73	1,88–3,97	**< 0,001**
	Baixa	15 (17,9)	33 (39,3)	36 (42,9)	1		

Determinantes intermediários em saúde							

Acesso a serviços de saúde							
Tipo de serviço médico	Público	62 (29,2)	67 (36,3)	73 (34,4)	2,37	1,09–5,16	0,029
	Privado	12 (48,0)	9 (36,0)	4 (16,0)	1		
Tipo de serviço odontológico	Público	30 (26,1)	48 (41,7)	37 (32,2)	1,29	0,81–2,06	0,291
	Privado	35 (47,2)	37 (30,6)	39 (32,2)	1		
Motivo da consulta odontológica	Rotina	46 (31,7)	47 (32,4)	52 (35,9)	1		
	Dor ou necessidade	29 (31,5)	39 (42,4)	24 (26,1)	0,80	0,50–1,30	0,300
Tempo da última consulta médica	Menos de 1 ano	71 (32,6)	77 (35,3)	70 (32,7)	1		
	1 vez ao ano ou mais	4 (20,0)	9 (45,0)	7 (35,0)	3,39	2,04–5,63	**< 0,001**

Comportamentos em saúde							
Escovação	Até 2 vezes/dia	21 (20,4)	34 (33,0)	48 (4,66)	2,91	1,78–4,76	**< 0,001**
	3 ou mais vezes/dia	54 (40,0)	52 (38,5)	29 (21,5)	1		
Fio dental	Usa diariamente	46 (60,5)	28 (36,8)	0 (0,0)	1		
	Não usa diariamente	29 (17,9)	58 (35,8)	75 (43,3)	9,16	5,13–16,37	**< 0,001**
Frequência/uso de serviço médico	1 vez/ano ou mais (uso regular)	57 (31,5)	63 (34,8)	61 (33,7)	1		
	- 1 vez/ano (uso irregular)	18 (31,6)	23 (40,4)	16 (28,1)	0,88	0,58–1,33	0,537
Frequência/uso de serviço odontológico	1 vez/ano ou mais (uso regular)	34 (58,6)	21 (36,2)	3 (5,2)	1		
	- 1 vez/ano (uso irregular)	39 (22,7)	63 (36,6)	70 (40,7)	5,77	1,95–17,10	**0,002**

Condições clínicas	Sem dor	45 (28,8)	53 (34,0)	58 (37,2)	1		
Dor (dentes/gengivas)	Alguma dor	30 (36,6)	33 (40,2)	19 (23,2)	0,61	0,37–1,01	0,054
Glicemia	Até 126 dmgl	38 (33,6)	40 (35,4)	35 (31,0)	1		
	127 dmgl ou mais	37 (29,6)	46 (36,8)	42 (33,6)	1,17	1,05–1,30	**0,006**
Hemoglobina Glicada (HbA1c)	Até 7,0%	35 (38,0)	27 (29,3)	30 (32,6)	1		
	7,1% ou mais	40 (27,4)	59 (40,4)	47 (32,2)	1,27	0,55–2,95	0,579
Pressão Arterial Sistólica	Até 139 mmhg	55 (31,6)	64 (36,8)	55 (31,6)	1		
	140 mmhg ou mais	20 (31,2)	22 (34,4)	22 (34,4)	1,08	0,90–1,29	0,424
Pressão Arterial Diastólica	Até 89 mmhg	65 (31,0)	80 (38,1)	65 (31,0)	1		
	90 mmhg ou mais	10 (35,7)	6 (21,4)	12 (42,9)	1,20	0,31–4,63	0,789
Bolsa periodontal (> 4mm)	Sim	62 (61,4)	39 (38,6)	0 (0,0)	0,39	0,01–0,47	0,039
	Não	13 (9,5)	47 (34,3)	77 (56,2)	1		
Biofilme dental visível	Sim	39 (48,1)	42 (51,9)	0 (0,0)	6,25	0,26–148,10	0,256
	Não	34 (21,9)	44 (28,4)	77 (49,7)	1		

Fonte: Elaboração dos autores (2020).

^a^ Teoria do Arco Reduzido (Armellini e Fraunhofer, 2004).

^b^ Salário mínimo (SM) brasileiro = R$ 998.00 (Dez / 2019).

No modelo final, as perdas dentárias foram associadas à maior idade (OR = 1,12; IC95% 1,07–1,17), menor escolaridade (OR = 3,43; IC95% 1,17–10,10), índices mais altos de glicemia (OR = 1,98; IC95% 1,00–3,92), não uso de fio dental (OR = 4,58; IC95% 1,75–7,31), uso irregular do serviço odontológico (OR = 2,60; IC95% 1,32–5,12), presença de bolsa periodontal > 4mm (OR = 0,31; IC95% 0,01–0,08) e biofilme dental visível (OR = 7,23; IC95%3,19–16,41) (Tabela 3).

**Tabela 3:** Modelos de regressão para a condição bucal perdas dentárias de indivíduos (n = 238) com doenças crônicas, usuários do serviço público de saúde de Piracicaba, SP, 2019.

Variáveis		Modelo 1			Modelo 2			Modelo 3			Modelo 4			Modelo 5			Modelo 6		
					
		OR	(IC95%)	p	OR	(IC95%)	p	OR	(IC95%)	p	OR	(IC95%)	p	OR	(IC95%)	p	OR	(IC95%)	p
**Idade**	Média em anos (62,7)	1,12	(1,08–1,16)	**0,000**							1,11	(1,08–1,15)	**0,000**	1,10	(1,06–1,14)	**0,000**	1,12	(1,07–1,17)	**0,000**
**Escolaridade**	Menos que 4 anos	6,52	(2,85–14,91)	**0,000**							5,55	(2,37–12,97)	**0,000**	3,18	(1,27–7,93)	**0,013**	3,15	(1,01–9,74)	0,046
	**Até 4 anos completos**	6,00	(2,80–12,85)	**0,000**							5,14	(2,35–11,25)	**0,000**	2,90	(1,22–6,93)	**0,016**	3,43	(1,17–10,10)	**0,025**
	5 anos ou mais	1									1			1			1		
**LS**	Baixa				4,70	(2,50–8,82)	**0,000**				1,86	(0,92–3,77)	0,083	1,45	(0,68–3,08)	0,326	0,93	(0,37–2,36)	0,894
	Média				2,80	(1,50–5,20)	**0,001**				1,67	(0,83–3,35)	0,146	1,84	(0,88–3,85)	0,104	1,59	(0,65–3,88)	0,308
	Alta				1,00						1			1			1		
**Uso de fio dental**	**Não usa diariamente**													3,58	(1,75–7,31)	**0,000**	4,88	(1,99–11,95)	**0,001**
	Usa diariamente													1			1		
**Frequência/uso de serviço odontológico**	uso regular (até 1 ano)				1									1			1		
	**Uso irregular (+ de 1 ano)**				2,60	(1,32–5,12)	**0,005**							1,94	(0,93–4,03)	0,075	3,15	(1,25–7,95)	**0,015**
**Tipo de serviço odontológico**	Privado				1														
	Público				1,38	(0,84–2,25)	0,196												
**Bolsa periodontal (> 4mm)**	Não							1									1		
	**Sim**							0,36	(0,01–0,07)	**0,000**							0,31	(0,01–0,08)	**0,000**
**Dor bucal**	Sem dor							1											
	Alguma dor							0,54	(0,30–0,96)	0,036									
**Biofilme dental visível**	Não							1									1		
	**Sim**							3,84	(2,09–7,04)	**0,000**							7,23	(3,19–16,41)	**0,000**
**Glicemia**	Até 126 dgml							1									1		
	**127 dgml ou mais**							1,50	(0,87–2,58)	0,138							1,98	(1,00–3,92)	**0,049**

Fonte: elaborada pelos autores (2020). Nota: **Modelo 1**: modelo de regressão com idade e escolaridade; **Modelo 2**: ajustado por tipo de serviço odontológico e LS; **Modelo 3**: ajuste para bolsa periodontal, dor dentes/gengivas, biofilme dental visível e glicemia; **Modelo 4**: ajustes dos Modelos 1 e 2 com escolaridade, LS e idade ; **Modelo 5**: ajustes dos Modelos 1, 2 e 3,escolaridade, LS, idade, frequência/uso odontológico e uso de fio dental; **Modelo 6**: ajustes dos Modelos 1, 2, 3 e 4 com escolaridade, LS, idade, frequência/uso odontológico, uso de fio dental, bolsa periodontal, biofilme dental visível e glicemia. Regressão logística multinomial.

## DISCUSSÃO

A LS foi fator associado significativo para a perda dentária, mesmo com os determinantes intermediários em saúde, acesso ao serviço e comportamentos em saúde, mas, quando ajustada por fatores sociodemográficos e condições clínicas, perdeu significância. Observou-se que conforme aumentam os gradientes de literacia em saúde, diminui-se o edentulismo. Porém, sabe-se que perdas dentárias sofrem influência de múltiplos fatores e, por isso, como demonstrado no modelo final desta análise, foi associada à idade, à escolaridade, ao não uso de fio dental, ao uso irregular de serviço odontológico, à presença de bolsa periodontal, ao biofilme dental visível e ao índice glicêmico. Destaca-se que, esses mesmos fatores, à exceção do índice glicêmico, também foram os fatores associados à LS, na análise bivariada.

A LS tem sido considerada um fator intermediário determinante nos comportamentos e desfechos em saúde^17^e fundamental nos dias atuais para compreensão das informações em saúde, o que acarreta em manutenção e recuperação da saúde^15^, conforme demonstram estudos recentes^18,42^. A associação com fatores intermediários de comportamento, como o uso regular de fio dental, o acesso ao serviço, a frequência e o tipo de serviço odontológico, também tem sido observada na literatura^11,43,44^, ratificando o papel da LS na tomada de decisão em saúde. Sendo assim, fica evidente que a LS é um determinante social importante a ser considerado como estratégia para se trabalhar na promoção da saúde e do bem-estar.

O uso de ajuste de modelos deste estudo pôde indicar que o principal desfecho em saúde bucal, a perda dentária, deriva de diversos fatores de risco acumulados ao longo da história do indivíduo e, por isso, os comportamentos de saúde, escovação dental, uso de fio dental e as condições clínicas, como as doenças bucais e gerais, condições mais proximais, acabam reduzindo o impacto da LS neste desfecho, quando esses aspectos são considerados. Porém observa-se que, conforme o aumento do gradiente literacia em saúde, aumenta também o edentulismo.

Nesse sentido, faz-se necessário demonstrar o papel intermediário da LS na redução de perdas dentárias, que é o principal resultado de saúde bucal, considerada um desafio global, e em doenças periodontais que, associadas às DCNT, têm consequências graves e impactam na qualidade de vida da pessoa^9^, embora ainda seja inconclusiva a associação entre perdas dentárias e a LS na literatura^18,45^.

Neste estudo, as perdas dentárias demonstraram associações com maior idade devido a um efeito de corte, ou seja, relacionado ao resultado histórico das políticas públicas em saúde bucal no Brasil e seu impacto na população^14^. A escolaridade, outro determinante primário em saúde, também foi associada às perdas dentárias, corroborando outros achados, em que menor escolaridade é associada a maior prevalência de perdas dentárias^46,47^, e estabelecida como indicador de risco em saúde^48^. Fatores sociodemográficos, econômicos e etários, encontrados também em outros estudos, são fatores estruturais e, portanto, difíceis de serem modificados^49,50^. A LS é um fator modificável e está fortemente associada a comportamentos e aspectos associados às perdas dentárias, embora sejam necessários outros estudos para esclarecer a associação com desfechos em saúde. A OMS destaca que a LS é peça chave para o desenvolvimento da promoção de saúde^15^, sendo também importante indicador de disparidades sociais^15^.

A variável renda foi associada à perda dentária na análise univariada, porém, no ajuste com escolaridade e idade, perde a significância (não foi associada à LS). Isso pode ter ocorrido devido à colinearidade com escolaridade e/ou à homogeneidade da amostra no aspecto socioeconômico onde a maioria tem renda de um salário mínimo ou mais e estudou até quatro anos.

Nesta amostra, foram encontradas associações de perdas dentárias com os comportamentos, como uso irregular de fio dental e uso de serviço odontológico, variáveis que impactam no aumento da prevalência da perda dentária em indivíduos com doenças crônicas, de acordo com estudo realizado em Santa Rita, Paraíba, Brasil^51^. Em relação às condições clínicas presença de biofilme dental e bolsa periodontal, também foram encontradas associações em demais estudos, reforçando a influência das DCNT sobre a doença periodontal, perda dentária e consequências sistêmicas^52,47^. Neste estudo, apresentaram maior presença de bolsa periodontal os que possuíam mais dentes presentes na boca, levando em consideração o grande número de edêntulos.

Os comportamentos são influenciados pelos determinantes e impactam nas doenças crônicas, por isso, dar atenção aos tipos de determinantes de iniquidades^53^pode determinar melhorias em saúde e nesse caso, a LS pode ser um agente modificador, empoderando o indivíduo e colocando-o como protagonista de sua saúde. Em suma, entender a LS associada a comportamentos em saúde torna-se um considerável preditor.

A associação entre alta glicemia e perdas dentárias, apontada neste estudo, comprova que a perda dentária é um indicador importante de saúde sistêmica, como também apontaram outros trabalhos^54,55^, sendo inclusive considerada como fator de risco para doenças cardíacas^12,56^ e para artrite reumatoide^57^. Além de potencializar condições mais graves de saúde, esta condição pode ser preditora de mortalidade^58^, fato de extrema relevância e que reforça a importância da abordagem da integralidade na saúde^59^.

Uma limitação deste estudo foi o recorte transversal, que impossibilitou uma inferência causal. Outro aspecto a ser considerado foi à homogeneidade da amostra, na qual todos os indivíduos apresentam doenças crônicas e possibilidade de comorbidades. Porém, a amostra é representativa dessa parcela da população que precisa ser estudada e inserida no serviço de saúde com olhar diferenciado e com maior consideração à saúde integral, já que é a faixa etária sobre a qual ocorre maior severidade de doenças bucais, principalmente a perda dentária^60^, seus determinantes podem causar reflexos na saúde sistêmica, portanto necessitando de rápida atenção.

Conhecer e identificar fatores de risco associados pode representar melhor manejo nos cuidados em saúde dessa população, bem como o desenvolvimento de estratégias de promoção da saúde. Uma vez que muitos sistemas de saúde no mundo não acompanham o aumento da carga de DCNT e as necessidades de saúde associadas da população^61^, este estudo mostra-se um relevante alerta para que gestores considerem a integralidade em saúde, bem como a LS, no trabalho de equipe multiprofissional^62^ e em suas ações de políticas públicas voltadas a essa população. Para tanto, também foi mostrado o uso de uma ferramenta de mensuração da LS de fácil aplicação para equipes de saúde atuarem no controle das DCNT.

Quando mensurável, por meio de instrumentos usados por profissionais de saúde (como o utilizado neste estudo), a LS também será importante para a modificação dos desfechos clínicos, podendo ser modificada por meio de intervenções e ações de promoção de saúde, aumentando a autonomia das pessoas nas tomadas de decisão. Com isso, é relevante considerar a integralidade em saúde e a LS nas políticas de promoção da saúde.

## CONCLUSÃO

A perda dentária esteve associada à literacia em saúde, quando ajustada por comportamentos em saúde, a partir da inclusão das variáveis sociodemográficas e condições clínicas, a literacia em saúde perdeu a significância. No modelo final, comportamentos, determinantes em saúde e condições clínicas foram indicadores de risco da perda dentária, demonstrando a multifatorialidade envolvida nesse fenômeno. Assim, sugere-se futuros estudos para compreender a perda dentária que abordem a literacia em saúde e integralidade.
